# Trapped in the epineurium: early entry into the endoneurium is restricted to neuritogenic T cells in experimental autoimmune neuritis

**DOI:** 10.1186/s12974-018-1259-5

**Published:** 2018-08-01

**Authors:** Anne K. Mausberg, Fabian Szepanowski, Francesca Odoardi, Alexander Flügel, Christoph Kleinschnitz, Mark Stettner, Bernd C. Kieseier

**Affiliations:** 1 Department of Neurology, Research Group for Clinical and Experimental Neuroimmunology, University Hospital Essen, Hufelandstr. 55, 45147 Essen, Germany; 2 Department of Neuroimmunology, University Medical Centre, Goettingen, Germany; 3 Department of Neurology, Medical Faculty, Heinrich Heine University Duesseldorf, 40225 Duesseldorf, Germany

**Keywords:** Neuritogenic T cells, Experimental autoimmune neuritis, Guillain-Barré syndrome, GBS, Chronic inflammatory demyelinating neuropathy, CIDP, GFP expression

## Abstract

**Background:**

Autoimmune polyneuropathies are acquired inflammatory disorders of the peripheral nervous system (PNS) characterized by inflammation, demyelination, and axonal degeneration. Although the pathogenesis has not been fully elucidated, T cells recognizing self-antigens are believed to initiate inflammation in a subgroup of patients. However, the route and time of T cell entry into the PNS have not yet been described in detail. In this study, we analyzed both kinetics as well as localization of retrovirally transfected green fluorescent protein (GFP)-expressing neuritogenic T lymphocytes in experimental autoimmune neuritis (EAN).

**Methods:**

T lymphocytes obtained from rats following EAN induction by immunization with peripheral nerve protein peptide P2_55–78_ were retrovirally engineered to express GFP. Non-specific T cells were negatively selected by in vitro restimulation, whereas GFP-expressing neuritogenic T cells (reactive to P2_55–78_) were adoptively transferred into healthy rats (AT-EAN). Antigen-specific T cell tracking and localization was performed by flow cytometry and immunohistochemistry during the course of disease.

**Results:**

After induction of autoimmune neuritis, P2-reactive T cells were detectable in the liver, spleen, lymph nodes, lung, peripheral blood, and the sciatic nerves with distinct kinetics. A significant number of GFP^+^ T cells appeared early in the lung with a peak at day four. In the peripheral nerves within the first days, GFP-negative T cells rapidly accumulated and exceeded the number of GFP-expressing cells, but did not enter the endoneurium. Very early after adoptive transfer, T cells are found in proximity to peripheral nerves and in the epineurium. However, only GFP-expressing neuritogenic T cells are able to enter the endoneurium from day five after transfer.

**Conclusions:**

Our findings suggest that neuritogenic T cells invade the PNS early in the course of disease. However, neuritogenic T cells cross the blood-nerve barrier with a certain delay without preference to dorsal roots. Further understanding of the pathophysiological role of autoagressive T cells may help to improve therapeutic strategies in immune-mediated neuropathies.

## Background

Autoimmune diseases of the peripheral nervous system (PNS) are characterized by mononuclear and T cell infiltration, demyelination, and axonal damage. The structural damage leads to functional impairment with sensory and motor deficits.

Autoimmune-mediated neuropathies comprise a broad spectrum of clinical disease, and different pathophysiological scenarios have been suggested. For the acute form—Guillain-Barré Syndrome (GBS)—molecular mimicry is thought to be the leading pathophysiological factor of the disease. Peripheral nerve epitopes resembling pathogen epitopes of a preceding infection, e.g., with *Campylobacter jejunii* [1], trigger an immune response against the PNS [2]. Myelin protein-specific autoagressive T cells are found in some GBS forms but also in chronic inflammatory demyelinating polyneuropathy (CIDP) [3]. Reactive T cells from patients with CIDP and GBS showed an increased proliferation and the cytokine production in response to peripheral myelin proteins. Oligoclonal expansion of T cells indicative for activation of the T cell repertoire has also be described in CIDP patients and suggests a pivotal role in disease mechanism [4–6].

The route and kinetics of neuritogenic T cells in inflammatory conditions of the PNS has not been understood in detail. Experimental autoimmune neuritis (EAN) induced in Lewis rats by myelin homogenates, or peptides of peripheral myelin components such as protein 2 (P2), is a well-defined animal model of a neuritis [7]. The adoptive transfer of neuritogenic CD4 T cells alone is sufficient to induce a comparable disease in the recipient animal [8]. Although this passive immunization model is well established, the fate of the neuritogenic T cells after transfer into a healthy rat has remained largely undefined. A better understanding of the fate of neuritogenic T cells after transfer in EAN may help to improve treatment strategies, specifically when treatment targets T cells.

We generated P2_55–78_-specific, neuritogenic T cells, which were retrovirally engineered to express green fluorescent protein. We were able to distinguish neuritogenic green fluorescent from endogenous polyclonal T cells after adoptive transfer. We analyzed the kinetics and distribution of neuritogenic T cells in the blood and various tissues including peripheral nerves.

## Methods

### EAN induction in Lewis rats

Animal experiments were approved by the local state authorities (Landesamt fuer Natur, Umwelt und Verbraucherschutz Nordrhein-Westfalen). Rats were housed under specific pathogen-free conditions in the animal research facility of the University of Duesseldorf.

To induce active EAN, female Lewis rats (8 weeks, Janvier, Le Genest-Saint-Isle, France) received subcutaneous injections of 200 μg of P2_55–78_ (JPT peptides, Berlin, Germany) in complete Freund’s adjuvant (CFA; BD, Heidelberg, Germany) containing heat-inactivated mycobacterium tuberculosis H37RA (2 mg/ml) (BD). A modified EAN score [9] was applied: 0 no impairment, 1 reduced tail tone, 2 limp tail, 3 absent righting reflex, 4 gait ataxia, 5 mild paraparesis, 6 moderate paraparesis, 7 severe paraparesis or paraplegia, 8 tetraparesis, 9 moribund, and 10 death due to neuropathy.

### Generation of T cell lines

CD4_P2-GFP_ cell lines were generated by isolation of cells from draining lymph nodes and restimulation with 10 μg/ml P2_53–78_ peptide 10 days after immunization. Three and 7 days after restimulation, T cell culture supplement with ConA (BD Bioscience, Germany) was added to the medium (RPMI 1640 with 5% FCS, 2 mM l-glutamine, 50 μM 2-ME, and non-essential amino acids, ThermoFisher, Darmstadt, Germany). Restimulated T cells were co-cultivated with the green fluorescent protein (GFP)-transduced packaging cell line GPE86 for retroviral transduction [10]. The packing cell line produces an ecotropic retrovirus during the first step of restimulation. Virus transduction resulted in allogenic expression of GFP and geneticin resistance in proliferating cells. Geneticin was added in the following three restimulation steps; thus, solely P2-specific, GFP-transduced T cells proliferated and survived. For restimulation, gamma-irradiated thymocytes (10 Gy, 1000 rad) were used as antigen-presenting cells. The P2-specific T cell line resulted in 98% GFP-expressing cells in flow cytometry analysis.

Adoptive transfer EAN was induced by intravenous injection of 5 × 10^6^ CD4_P2-GFP_ cells per animal at day three after the last restimulation. Neuritogenic T cells without GFP expression (CD4_P2_) served as a control.

### T cell proliferation assay

Draining lymph nodes of rats 10 days after immunization were dissected under sterile conditions and passed through a 40-μm cell strainer. The derived cell suspension was cultured in flat bottom 96-well plates in standard T cell medium (RPMI 1640 with 5% FCS, 2 mM l-glutamine and 50 μM 2-ME, life technologies, ThermoFisher). P2_53–78_ was added to each well in concentration from 0 to 100 μg/ml. T cell proliferation was measured via [3H] thymidine incorporation during the last 24 h of a 3-day incubation. Results from liquid scintillation counting (BetaPlate1205, Perkin Elmer, Boston, USA) are given as mean counts per minute (cpm) of quadruplicate cultures ± SEM. A stimulation index was calculated as the ratio of cpm at indicated P2_53–78_ concentrations to proliferation in the absence of antigen.

### Immunofluorescence

At indicated time points after AT, animals were sacrificed and perfused first with PBS and then with paraformaldehyde. Organs were embedded in Tissue-Tek (Sakura, Netherlands) and frozen at − 80 °C. Ten-micrometer sections (Cryostat CM1950 Leica, Wetzlar, Germany) were stained with mouse anti-rat CD4 antibody (Immunotools, Friesoythe, Germany) and rabbit anti-GFP antibody (ThermoFisher) using biotinylated- or fluorescent-conjugated secondary antibodies (goat anti-rabbit and horse anti-mouse, Dye488 and Dye633, Vector, USA). Slices were covered using Vectashield (Vector) mounting medium containing 4,6′diamidino-2-phenylindole (DAPI). For quantification, three longitudinal sections per animal were photographed with a high magnification (Axioplan 2, Zeiss). Cells were counted manually by a blinded investigator using Fiji/ImageJ 1.46j software (NIH, Bethesda, USA) and adjusted to the analyzed area.

### Cell extraction

Anesthetized rats were transcardially perfused with 50-mL ice-cold PBS. Cell extraction from sciatic nerve was performed as previously described [11], slightly adapted for the rat model. Briefly, sciatic nerves were homogenized in DMEM with 5% FCS using a scalpel and incubated with 1 mg collagenase/dispase (Roche, Mannheim, Germany) and 100 μg DNase I (Roche) at 37 °C for 45 min each. Cells were washed twice with DMEM containing 5% FCS, resuspended in cold medium, and passed through a 70-μm cell strainer. Cells were centrifuged on a 30%/70% percoll gradient (GE healthcare, Freiburg, Germany) at 1000*g* for 30 min. Nerve mononuclear cells were collected from the interphase and washed in culture media. Lung and liver tissue was treated analogously. Lymph nodes and spleen were homogenized and passed through a 40-μm cell strainer. Erythrocytes were lysed using Pharmlyse (BD Biosciences) according to the manufacturers’ protocol before subsequent flow cytometry.

### Flow cytometry

Cells were surface-stained using antibodies recognizing PE-labeled mouse anti-rat CD3 (clone G4.18), APC-labeled mouse anti-rat CD4 (OX-35), PerCP-labeled mouse anti-rat CD8a (OX-8), and APC-Cy7-labeled mouse anti-rat CD45 (OX-1) according to standard procedures. GFP fluorescence was detected in the FITC channel. Fluorescence was measured and analyzed on a FACSCanto II flow cytometer (BD Biosciences). Distinct cell populations were analyzed using BD FACSDiva 8.0 and FlowJo 10.0 (LCC, Oregon, USA).

### Data analysis

Data were analyzed using GraphPadPrism 5.0 (GraphPad Software, La Jolla, US). The Wilcoxon-Mann-Whitney test was used to determine statistically significant differences in clinical score values. Student’s *t* test for unrelated samples was used to determine statistically significant differences in all other analyses. Differences were considered significant at *p* values < 0.05.

## Results

### GFP expression of neuritogenic T cells did not reduce EAN severity or alter infiltrating populations after adoptive transfer

Lymph node cells of immunized rats were restimulated with P2 peptide in the presence of the GFP packaging cell line GPE-86 to retrovirally transduce P2-recognizing and P2-proliferating T cells. This retrovirus also contained the geneticin resistance gene. Successful induction of immunization was routinely measured in a T cell proliferation assay (data not shown). Selection with geneticin in the following three stimulation rounds resulted in a subsequent enrichment of GFP-expressing P2-specific T cells. The last final restimulation was performed in the absence of geneticin to avoid antibiotic side effects. The way of restimulation (exogenously added peptide and the sequence of chosen antigen) favors CD4 T cell expansion, and the resulting T cell lines were all CD4 positive.

**Fig. 1:**
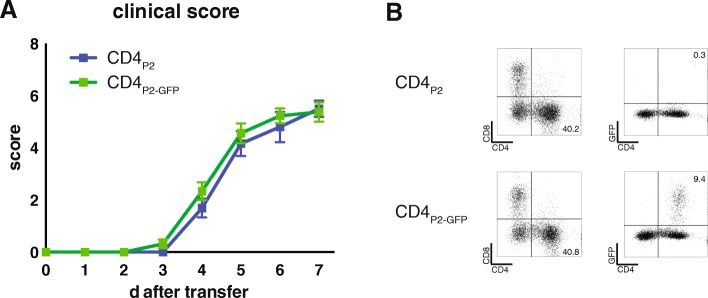
No differences of EAN disease course and T cell infiltration of the sciatic nerve with neuritogenic T cells expressing GFP or non-GFP-expressing control T cells. **a** Clinical course of the disease of animals used for the analysis of adoptive transfer of CD4_P2-GFP_ T cells. Three independent experiments with *n* = 2–4 rats at each time point (*n* = 59 in total). Neuritogenic T cells without GFP expression served as a control (*n* = 10). Clinical scoring was assessed daily using the following system: 0 no impairment, 1 reduced tail tone, 2 limp tail, 3 absent righting reflex, 4 gait ataxia, 5 mild paraparesis, 6 moderate paraparesis, 7 severe paraparesis or paraplegia, 8 tetraparesis, 9 moribund, and 10 death due to neuropathy. Depicted are clinical score means ± SEM for each day. At no time point, a statistical significance was detected (*p* > 0.5; Wilcoxon-Mann-Whitney test). **b** Gated on lymphocytes, frequencies of T cells were determined after adoptive transfer of GFP-transduced (CD4_P2-GFP_) and non-transduced neuritogenic control cells (CD4_P2_) in the sciatic nerve at day seven. Representative flow cytometry staining of CD4 and CD8 T cells gated on CD45^+^ cells are shown on the left side. Upper panel shows T cell composition after transfer of CD4_P2_ control cells; lower panel shows the results of GFP-expressing neuritogenic T cells (CD4_P2-GFP_). Numbers indicate the percentages of CD4 T cells within the lymphocyte gate. On the right, representative dot plots from CD4 staining and GFP expression (measured in FITC channel) gated on CD45^+^ cells are shown. Numbers indicate the percentages of GFP^+^ T cells within the lymphocyte gate

T cell lines were used for experiments, when a minimum of 95% of resulting neuritogenic T cells expressed GFP (CD4_P2-GFP_). To ensure that GFP expression did not reduce T cell neuritogenicity, T cells without GFP expression vector were used as controls (CD4_P2_). These T cells were treated as CD4_P2-GFP_ T cells, but the packaging cell line did not contain the GFP-expressing vector. Injection of T cell lines CD4_P2_ and CD4_P2-GFP_ resulted in a comparable clinical score. The passive EAN induction was independent of GFP expression. The first clinical signs were observed at days three or four after treatment, the maximum was reached by days five to seven. In most of the animals, no clinical signs were observed until day three (Fig. 1a). At day seven after transfer, the sciatic nerves of the animals were prepared and T cell composition of infiltrating cells was determined via flow cytometry (Fig. 1b). In the depicted representative dot plots, the distribution of CD4 and CD8 T cells was demonstrated (left panel) after transfer of CD4_P2_ (upper panel) and CD4_P2-GFP_ T cells (lower panel). Numbers in the lower right quadrant represent percentages of CD4 T cells within the leukocyte gate of the sciatic nerve. Seven days after adoptive transfer, predominantly CD4 T cells were found in the nerve, while CD8 T cells were not as frequent. The percentage varied between different animals but the number of infiltrating T cells within the sciatic nerve did not correlate with the clinical score (data not shown). The percentage of GFP-expressing T cells is shown in Fig. 1b, right panel. Here, CD4 T cells and GFP expression are depicted. Numbers in the upper right quadrant represent percentages of CD4 T cells that express GFP.

The adoptive transfer of CD4_P2_ control T cell lines resulted in CD4^+^GFP^+^ cells below 0.5%. In sciatic nerves of animals receiving CD4_P2-GFP_, T cells vary from 4.5 to 11.4% of lymphocytes. No GFP expression was detected in non-CD4 T cells (lower left panel) within the lymphocytes. Again, no correlation of the clinical score with infiltrating T cells or GFP-expressing T cells was detected (data not shown).

### Route and kinetics of neuritogenic T cells within the organism after adoptive transfer

**Fig. 2:**
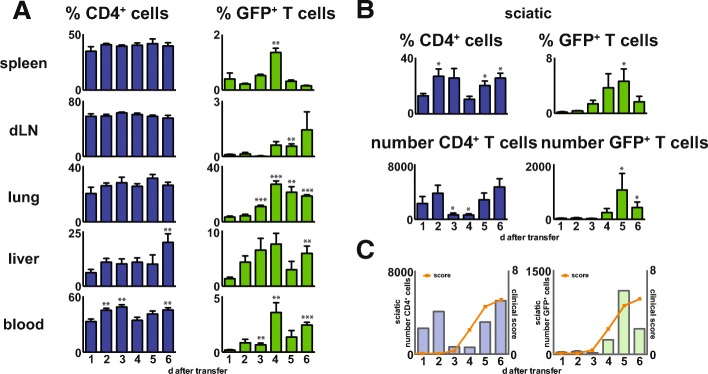
Flow cytometry analysis of kinetics of GFP-expressing neuritogenic T cells in different organs after AT-EAN. Flow cytometry analysis of the percentages of T cells (blue panel) and GFP-expressing neuritogenic T cells (green panel) in the different organs at the indicated time points (days one to six) after adoptive transfer of CD4_P2-GFP_ gated on lymphocytes. Number of T cells was calculated based on the overall number of lymphocytes in the sciatic nerve determined by flow cytometry. GFP expression was detected in FITC channel. **a** Percentage of T cells and neuritogenic GFP-expressing in the spleen, draining lymph node (dLN), lung, liver, and blood. Depicted is mean ± SEM. Asterisks indicate significant differences compared to day one (*p < 0.05, **p < 0.01, ***p < 0.001; Student’s *t* test). **b** Percentage and number of T cells and neuritogenic GFP-expressing in the sciatic nerve. Note that the preparation method of the sciatic nerve cannot differentiate between endoneurium, perineurium, and epineurium. Depicted is mean ± SEM. Asterisks indicate significant differences compared to day one (*p < 0.05, **p < 0.01, ***p < 0.001, Student’s *t* test). **c** Percentage of T cells (blue bars) and GFP-expressing neuritogenic T cells (green bars) on the left *y* axis and the clinical score (orange) on the right *y* axis at the indicated time points (days one to six). To simplify the graph, only the mean is depicted

Clinical signs of neuritis were observed only after 3 to 4 days post-adoptive transfer of neuritogenic cells. Immunologically relevant organs (spleen, blood, sciatic nerve draining lymph node), the lung, and the liver as well as the sciatic nerve were prepared daily from day one to day six after adoptive transfer of CD4_P2-GFP_, and the proportion of CD4 T cells (blue bars) and GFP-expressing T cells (green bars) was determined (Fig. 2). Frequencies of T cells were stable at around 30 to 60%, in the spleen, LN, lung, and in the peripheral blood during the adoptive transfer, while T cells in the liver were less frequent (approximately 12%) until day five and were slightly higher at day six (Fig. 2a). While CD4 frequencies do not allow a discrimination between P2-specific and non-specific T cells, expression of GFP is indicative for neuritogenic CD4_P2-GFP._ In the periphery, we detected few GFP-expressing T cells in the spleen, the sciatic nerve draining lymph nodes (dLN), and in the blood, where GFP T cells were almost not detectable at days one to three but doubled at days four to six. Interestingly, the number of GFP T cells in the lung was increased at day four (around 30%), whereas before this was below 10%.

In the liver, the GFP T cell number increased up to day four, and then from days five and six the numbers decreased to the same level as in the lung. In the sciatic nerve, a limited number of T cells was detectable (below 10% after preparation with our protocol, data not shown) and passive immunization with CD4_P2-GFP_ T cells resulted in a CD4 T cell increase up to 30% by day three and then a decreases to the baseline with a secondary increase to 30% at day six (Fig. 2b). Compared to this, the proportion of CD4_P2-GFP_ is limited to a small fraction. At days one and two, almost no CD4_P2-GFP_ can be detected in the nerve, from days tree to five arise of up to 5%, while single animals revealed percentages up to 19.3%, and then again decreasing from day six. While T cell numbers were more or less stable in the analyzed compartments and do not vary over time, T cell numbers and infiltrating cells in general in the sciatic nerve differed tremendously during adoptive transfer experiments.

Since relative frequencies do not indicate the absolute changes in T cell numbers, CD4 T cell numbers are also depicted (Fig. 2b). In the sciatic nerve of a healthy rat, the preparation method in our hands resulted in approximately 1000 leukocytes from a healthy nerve, of which approximately 10% are CD4% T cells (data not shown). At days one and two after adoptive transfer, the number of leukocytes increased to 3000 per sciatic nerve, but decreased at days three and four to baseline values. At days five and six in a second increase to approximately 4000, T cells were detectable within the nerve. Interestingly, the GFP T cell numbers revealed a completely different kinetic. At days one to three, almost no GFP^+^ T cells were detectable and thereafter an increase to day five of up to 1000 GFP T cells. At day six, less GFP T cells were obtained from the whole sciatic nerve preparations. To visualize the kinetic of T cell infiltration in comparison to the progression of clinical signs, we combined the graph showing the amount of T cells in the sciatic nerve with the graph of the clinical score (Fig. 2c). While CD4 T cells infiltration was detectable as a double peak independent of the clinical score, GFP T cell infiltration showed similarities to the clinical score.

### Number of neuritogenic T cells in the sciatic nerve endoneurium after adoptive transfer

**Fig. 3:**
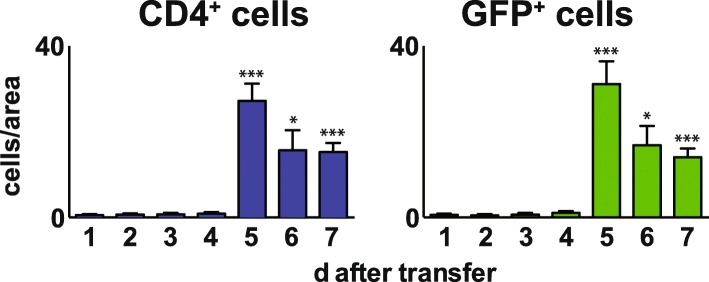
Kinetics of GFP-expressing neuritogenic T cells in the endoneurium after AT-EAN. Histological analysis of the number of T cells (blue) and GFP-expressing neuritogenic T cells (green) in the sciatic nerve endoneurium after adoptive transfer of CD4_P2-GFP_ at the indicated time points. T cells were counted in cross sections of five random images in four different regions (from proximal to distal) of sciatic nerves from animals of independent experiments (*n* = 4). Cross sections were stained with antibodies against either CD4 or GFP. Cell nuclei were visualized using DAPI. Note that the number of GFP-positive cells is as high as the number of CD4 T cells (GFP vs. CD4 *p* > 0.5 for each time point, unpaired Student’s *t* test). Representative images used for counting are shown in Fig. 4a. Depicted are mean positive cells/area ± SEM. Asterisks indicate significant differences compared to day one (*p < 0.05, **p < 0.01, ***p < 0.001, Student’s *t* test)

In contrast to flow cytometry results for T cells from whole nerve homogenates, immunohistochemistry analysis allows distinct localization of T cells within the peripheral nerve. Flow cytometry suggested two waves of invasion of unspecific T cells at days one to two and a second increase at days five to six. Using immunohistochemistry (Fig. 3), almost no T cells were detected in the endoneurium before day four but thereafter followed a massive increase at day five, which fell rapidly by one half at days six and seven. Most interestingly, these numbers were almost congruent between CD4^+^ and GFP^+^ T cells, indicating that all CD4 T cells that entered the endoneurium express GFP. These cells were neuritogenic T cells specific for the P2 peptide.

### Localization of neuritogenic T cells in the sciatic nerve after adoptive transfer

**Fig. 4:**
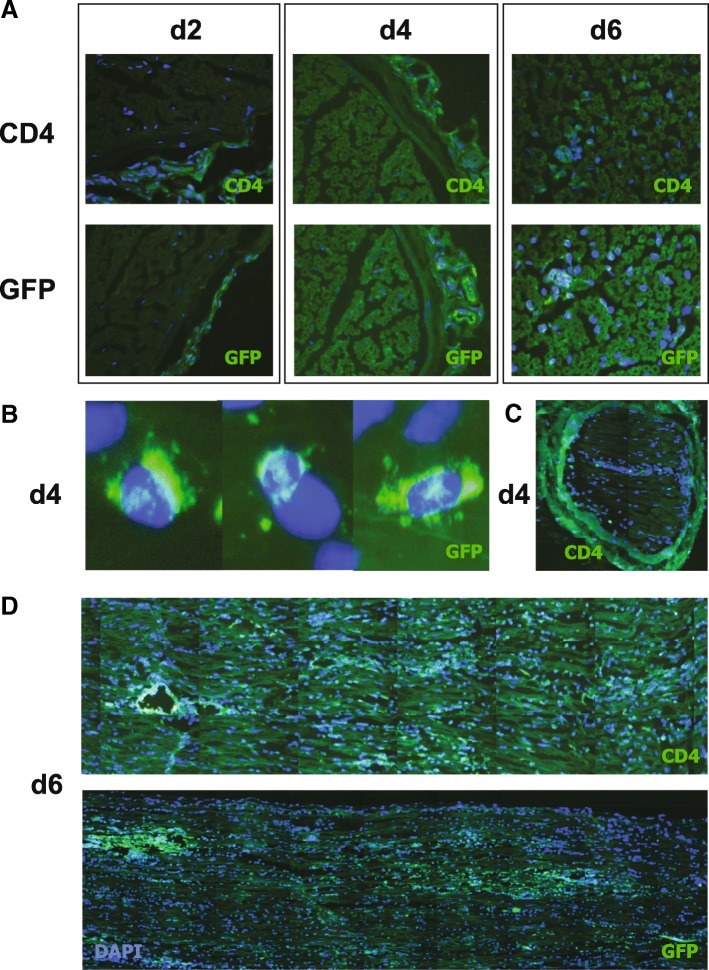
Distribution of GFP-expressing neuritogenic T cells in the sciatic nerve after AT-EAN. Histological analysis of distribution of GFP-expressing neuritogenic T cells after adoptive transfer of CD4_P2-GFP_ at different time points in the sciatic nerve. **a** Cross sections stained with antibodies against either CD4 (upper panel) or GFP (lower panel). Cell nuclei were visualized using DAPI. Representative images of days two and four both showed endoneurium and epineurium. Note the massive cell infiltration in the epineurium, while no cells are detectable in the endoneurium. Endoneurium is visualized for day six. **b** Higher magnification of GFP^+^ cell in the epineurium at day four. **c** Overview of cross sections stained with antibodies against CD4. Representative image of a rare example where CD4 T cells (and GFP^+^ T cells, data not shown) are detectable in the endoneurium at day four. **d** Representative longitudinal sections of a proximal region stained with a CD4 antibody (upper panel) or a distal region with GFP antibody (lower panel) at day six. Cell nuclei were visualized using DAPI. Note the patchy cell infiltration in the endoneurium

Immunohistochemistry staining with CD4 and GFP antibodies of cross sections of sciatic nerves at the indicated time points revealed that T cells as well as the GFP-expressing cells were localized mostly in the epineurium (Fig. 4a). Six days after transfer, CD4- and GFP-positive cells were found in the endoneurium, and almost exclusively neuritogenic T cells enter the endoneurium (Fig. 4b). Cells accumulated in proximity to epineural blood vessels. Representative images of a cross-sectional staining of CD4 at day four revealed few cells in the endoneurium, while most of the cells are epineurally localized (Fig. 4c), GFP-staining revealed comparable results (data not shown).

Longitudinal sections of the sciatic nerve revealed no distinct distribution of CD4- and GFP-expressing T cells concerning distal or proximal localization in the nerve, neither in the early phase of the adoptive transfer nor at later time points (Fig. 4d). No obvious predisposition for a distal or proximal infiltration of the nerve could be detected. The above-mentioned kinetic results were confirmed in the longitudinal sections.

## Discussion

In recent years, a significant progress has been reported to define the entry sites of encephalitogenic T cells into the central nervous system (CNS). In comparison, the pathomechanism of PNS infiltration in immune-mediated neuropathy is poorly understood.

Here, we used a retroviral approach to generate GFP-expressing neuritogenic P_2_-specific T cells. Passive immunization with these T cells resulted in the induction of EAN. We confirmed that expression of GFP reduces neither the neuritogenic capacity of the cells nor the T cell infiltration into the peripheral nerve. Neuritogenic T cells expressing GFP are as potent as non-GFP-expressing neuritogenic cells. The adoptive transfer of neuritogenic resulted in a comparable clinical score, but a shifted clinical course with a delayed infiltration pattern at time points varying between 1 and 2 days in different experiments. Despite the slight heterogeneity of the experiments, we minimized this problem by using adoptive transfers that resulted in a comparable clinical course, maximum score, and onset of the disease.

The distribution of T cells may argue for the following migration pattern of GFP-expressing neuritogenic T cells: T cells injected intravenously first enter the lung, the liver, and the spleen. Such a distribution within the first days of adoptively transferred cells is described for the injection of TCR transgenic T cells [12]. We observed a significant increase in the number of T cells in the lung starting at day three. Interestingly, these findings are in line with observations of encephalitogenic T cells by Odoardi et al. [13]. While Pape et al. interpreted the data as an observation of pure statistical distribution, it is well known that the lung is an important step to enhance the migratory capacity of T cells in search of their cognate antigen and therefore full activation of the cell [13]. Interestingly, the antigen-specific T cells must enter the lung to develop a migratory capacity after adoptive transfer, but homing into the lung is not restricted to encephalitogenic T cells. Neuritogenic T cells in the liver most likely are undergoing apoptosis [14]. Notably, in the draining lymph nodes of the sciatic nerve, and also in the sciatic nerve, neuritogenic T cells are almost absent before day three after transfer. From days three to five, neuritogenic T cells then enter their target tissue. They can be found in the draining lymph nodes and progress from the epineurium to the endoneurium of the sciatic nerve.

In contrast to that, endogenous non-neuritogenic T cells revealed a double wave of T cell infiltration. The first wave begins at day one and peaks at day three, and the second wave starts with after decline at day four with a second peak at days five and six. The two-wave kinetic was at first puzzling; however, immunohistochemistry resolved the problem. Analyzing the cells of the endoneurium, we found that the two waves of endogenous T cells in the sciatic nerve do not enter the endoneurium they accumulate at the epineurium without actually crossing the blood-nerve barrier. Interestingly, only neuritogenic T cells are able to enter the endoneurium at an early stage of the disease up until day seven.

The clinical manifestation of the neuropathy can be observed before the massive wave of specific T cells has reached the endoneurium of the nerve by day five. The numerous T cell accumulation of the epineurium and probably a few cells in the endoneurium are sufficient to disturb the PNS homeostasis.

Significant gaps in the understanding of the route and kinetics of neuritogenic T cells in inflammatory conditions of the PNS remain. For inflammatory conditions of the CNS, it has been shown that T cells are able to overcome the BBB in three steps [15]. In the first phase, T cells arrest at the walls of leptomeningal vessels and patrol the luminal surface. After diapedesis, they search the abluminal vascular surfaces and leptomeningal membrane for antigens. If they encounter a phagocyte presenting a recognized antigen, the T cells become activated and produce inflammatory mediators and cytokines and tissue infiltration is initiated [15]. The route of T cells migrating into their target tissues is a complex orchestration of different adhesion molecules, proteinases, and chemokines. In the nervous system, this mechanism is well regulated, since T cells must transgress the BBB in the CNS or the BNB in the PNS. In the CNS, possible entry sites are restricted to specialized compartments. [16]. Our data suggests that for the PNS, neuritogenic T cells have no predominant entry gate and no preference towards distal or proximal infiltration of the nerve in EAN. The patchy infiltration of the cells rather suggests a perivascular entry of autoimmune T cells into the endoneurium and macrophages, either endoneural or hematogenous [17] and/or Schwann cells [18, 19], could serve as antigen-presenting cells.

## Conclusions

We demonstrated that T cells enter the PNS in two waves after adoptive transfer of neuritogenic T cells. The first wave resulted in an accumulation of endogenous non-neuritogenic T cells in close proximity to nerve fibers. In the second wave, starting at day four after transfer, endogenous and neuritogenic T cells accumulate close to nerve fibers, but only the neuritogenic T cells were found in the endoneurium. Taken together, our data indicate that the actual crossing of the blood-nerve barrier is restricted to antigen-specific T cells in the early phase after disease induction.

## Abbreviations

BBB: Blood brain barrier
BNB: Blood-nerve barrier
CFA: Complete Freund’s adjuvant
CIDP: Chronic inflammatory demyelinating polyneuropathy
CNS: Central nervous system
DAPI: 4′,6-Diamidin-2-phenylindole
dLN: Draining lymph node
EAN: Experimental autoimmune neuritis
GBS: Guillain-Barré syndrome
GFP: Green fluorescent protein
P2: Peripheral myelin protein 2
PNS: Peripheral nervous system
